# The role of DC subgroups in the pathogenesis of asthma

**DOI:** 10.3389/fimmu.2024.1481989

**Published:** 2024-10-28

**Authors:** Jiangang Xu, Shuxian Cao, Youhua Xu, Han Chen, Siji Nian, Lin Li, Qin Liu, Wenfeng Xu, Yingchun Ye, Qing Yuan

**Affiliations:** ^1^ School of Basic Medical Sciences, Public Center of Experimental Technology, Southwest Medical University, Luzhou, Sichuan, China; ^2^ School of Stomatology, Southwest Medical University, Luzhou, Sichuan, China

**Keywords:** asthma, DC, DC subgroups, pathogenesis, asthma treatment

## Abstract

Dendritic cells (DCs), specialized antigen-presenting cells of the immune system, act as immunomodulators in diseases of the immune system, including asthma. The understanding of DC biology has evolved over the years to include multiple subsets of DCs with distinct functions in the initiation and maintenance of asthma. Moreover, most strategies for treating asthma with relevant therapeutic agents that target DCs have been initiated from the study of DC function. We discussed the pathogenesis of asthma (including T2-high and T2-low), the roles played by different DC subpopulations in the pathogenesis of asthma, and the therapeutic strategies centered around DCs. This study will provide a scientific theoretical basis for current asthma treatment, provide theoretical guidance and research ideas for developing and studying therapeutic drugs targeting DC, and provide more therapeutic options for the patient population with poorly controlled asthma symptoms.

## 1 Introduction

Asthma is the most common chronic lung disease and can be classified as allergic or nonallergic asthma in the presence or absence of defined allergens. Most asthma cases are type 2 (T2) allergic asthma caused by sensitization to innocuous environmental allergens, including house dust mites (HDMs), pollen, and animal dander, and are characterized by eosinophilia, high levels of IgE and fractional exhaled nitric oxide (FeNO), and high levels of Th2-type cytokines (1, 2). T2-high asthma is usually treated with glucocorticoids and biologics that target Th2-type cytokines and related pathway molecules (3). However, specific disease indications for patients with T2-low asthma are absent due to the lack of clarity of the inflammatory markers (4). Additionally, patients with this type of asthma are generally glucocorticoid-resistant and poorly responsive to pharmacologic therapy. To summarize, the mechanism underlying T2-low asthma is complex and variable and needs to be further elucidated.

At an early stage, DCs serve as the most potent and effective APCs in the body. They are important for mediating the innate immune response and inducing adaptive immune responses (5). Notably, DCs regulate the Th1, Th2, and Th17 immune responses and induce regulatory T cells (Tregs) that mediate tolerance (6, 7). In patients with asthma, DCs detect allergens and present antigens, which subsequently induce downstream innate asthma. This induces a downstream differentiation bias of naïve T lymphocytes involved in the development of asthma. With more information on DC subpopulations, the mechanism underlying the effects of different DC subtypes on asthma has been elucidated.

Here, this review summarizes the roles of different DC subgroups in asthma pathogenesis and DC-related asthma therapeutic studies, hoping to add further understanding of asthma pathogenesis as well as provide scientific theoretical guidance and future research ideas for asthma therapy and drug development targeting DCs.

## 2 Introduction to DCs

### 2.1 Biological development of DCs

**Figure 1:**
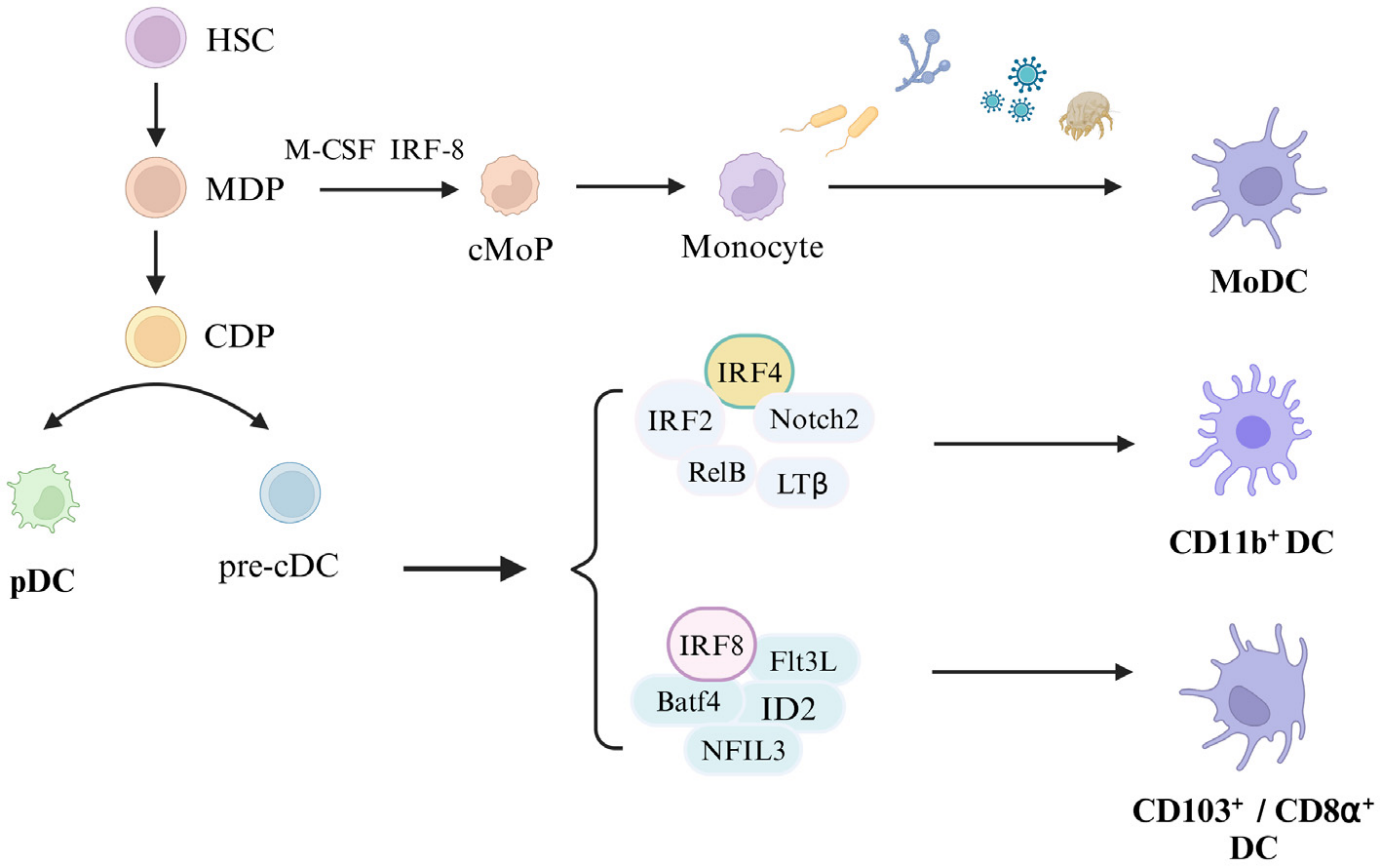
Development of Dendritic cells. Hematopoietic stem cells (HSC) differentiate into monocytes and dendritic cell progenitors (MDP), and MDP develop into common dendritic cell progenitors (CDP). Monocyte dendritic cell progenitors (MDP) eventually develop into monocytes under the stimulation of macrophage colony-stimulating factor (M-CSF) and IRF-8, and monocytes differentiate into monocyte-derived dendritic cells (MoDC) upon the stimulation with external substances (including bacteria, fungi, viruses, and allergens). CDP differentiates into plasmacytoid dendritic cells (pDC) and classical DC precursor cells (pre-cDC), and pre-cDC differentiates into CD11b^+^DC, CD103^+^DC and CD8α^+^DC under certain conditions. Among them, CD11b^+^DC development depends on IRF4, IRF4, Notch2, RelB and LTβ, and CD103^+^ DC or CD8α^+^DC development relines on IRF8, Batf4, Flt3L, ID2, and NFIL3.

The biological development of dendritic cells (DCs) has been extensively covered in recent literature, including comprehensive reviews by Cabeza-Cabrerizo et al. (8). However, it is important to note that the current review does not aim to focus on the developmental aspects of DCs, which will be depicted graphically for clarity (**Figure 1**).

### 2.2 Biological characterization and function of DC subpopulations

Dendritic cells are a heterogeneous family of myeloid antigen-presenting cells composed of several distinct subpopulations. They are found throughout the body, and although the number of DCs in any given tissue is only 2–4% of all leukocytes, the composition of the different subsets varies between organs. The tissue localization, expression of surface membrane proteins, and functions of the cells define these DC subpopulations. Typically, DCs are classified as pDCs, which can produce large amounts of IFN-I in response to viral infections, and cDCs, which play a highly efficient APC role in activating naïve T cells (9). Based on the differences in development, marker expression, and functional capacity, cDCs can be further classified into Type 1 classical dendritic cells (cDC1) and Type 2 classical dendritic cells (cDC2) (10). Refer to **Table 1** for a comprehensive overview of the classification and identification criteria for DC subgroups.

**Table 1:** Phenotypic markers of DC subsets.

Species	Marker	cDC1	cDC2	pDC	MoDC
Mouse	Clec9a	+	-	-	-
	XCR1	+	-	-	-
	SIRP-a(CD172a)	-	+	+	+
	CD45RA	-	-	hi	-
	CD3	-	-	-	-
	CD19	-	-	-	-
	NK1.1	-	-	-	-
	Gr-1	-	-	-	-
	CD8α	+	-	-	-
	CD103	+	Tissue- dependent	-	-
	CD11c	hi	hi	int	+
	MHCII	hi	+	lo	+
	CD11b	-	hi	-	+
	B220 (CD45R)	-	lo	+	-
	Ly6C	-	?	hi	int
	Ly49Q	-	-	hi	-
	Siglec-H	-	-	hi	-
	PDCA1 (CD317)	-	-	+	-
	CD64	?	-	-	int
	CCR2	-	?	-	+
	CD209	-	-	-	+
Human	HLA-DR	+	+	+	+
	CD11c	+	hi	lo	+
	CD123	-	-	+	-
	CD11b	-	+	-	+
	Sirp-α (CD172)	-	+	-	+
	CD141	+	-/+	-	+
	Clec9A	+	-	-	-
	CD1c	-	+	-	+
	BDCA2 (CD303)	-	-	+	-
	BDCA4 (CD304)	-	-	+	-
	CD45RA	-	-	+	?
	CD14	-	-	-	int
	CD206	-	-	-	+

Expression levels of cell surface markers: "+" (positive), "-" (negative), "-/+" (weakly positive/variable), "int" (intermediate), "hi" (high), "low" (low), "?" (unknown/variable). Markers are based on selected references (6, 11–16).

#### 2.2.1 cDC

##### 2.2.1.1 cDC1

The cDC1 subpopulation specifically expresses the C-type lectin receptors Clec9a and XCR1, whereas cDC2 expresses Sirpα (CD172α). However, cDC1 and cDC2 also express other markers. Unlike pDCs, cDCs may be accompanied by CD45RA, CD45R, or CD317 (PDCA1) markers (11). Additionally, CD26 markers may help identify mature cDCs expressing low levels of CD11c (12). When the internal environment of the body is in homeostasis, mouse cDCs have high levels of CD11c and MHCII expression, but T cells, B cells, NK cells, and granulocyte lineage markers (CD3, CD45R, CD19, NK1.1, and Gr-1) are absent (11). The identification of cDC1 in mice is generally based on the expression of MHCII, CD11c, and CD8a or CD103, as well as, the absence of CD11b and B220. CD103, a surface marker, is expressed not only in cDC1 but also in some tissues, such as intestinal tissues, as well as, in the cDC2 subpopulation (17). Second, CD8α is usually expressed on DCs in lymphoid organs, including the spleen and other lymphoid organs. It is commonly used as a characteristic marker of lymphoid-resident DCs (18).

In humans, the counterpart of cDC1 in mice is CD141^+^DC or blood DC antigen 3 DC (BDCA3^+^ DC). These cells constitute the DC subpopulation of about 0.03% of human PBMCs. The cell surface markers that define human cDC1s include HLA-DR^+^ CD11c^+^ CD123^-^ CD11b^-^ Sirpα^-^(CD172^-^) CD141^+^ Clec9A^+^ (11).

##### 2.2.1.2 cDC2

Mouse cDC2s are defined as CD11c^hi^ CD45R^lo^ MHCII^+^ CD8^-^ CD11b^+^ Sirpα^+^. However, as this subgroup is highly heterogeneous, it can also be further subdivided into Esam^hi^ CD8^–^ DCs and Esam^lo^ CD8^–^ DCs based on the two markers, Clec12A and Esam (19, 20). In humans, cDC2s are CD1c^+^ DCs with a biological signature that differs from that of mice. CD11b is a crucial surface marker in mouse cDC2s; however, unlike in mice, CD11b is not expressed by human cDC2s (12). Human CD1c cell surface markers can be defined as HLADR^+^ CD11c^hi^ CD123^-^ Sirpα^+^ CD1c^+^ Clec9A^–^. Thus, CD1c is a characteristic marker of CD1c^+^ DCs in humans, whereas cDC2s in mice do not express this marker. The expression of human CD1c is not limited to CD1c^+^ DCs but is also expressed in B cells and other DC subpopulations (e.g., CD141^+^ DCs and monocytes).

Different cDC subtypes not only express slightly different surface markers, but they also differ in their biological functions. Lung-resident cDC1s effectively mediate CD8^+^ T-cell activation through cross-presentation, generating a Th1-type cellular immune response against viral and bacterial pathogens (10, 21). Specifically, cDC1s take up exogenous antigens, which are processed to form MHCI antigenic peptide complexes and are ultimately presented to CD8^+^ T cells, thus triggering the entry of an intracellular pathogen and the killing of tumors with intracellular pathogens. Additionally, cDC1s are commonly associated with tolerance functions (22). In contrast, cDC2s, which are associated with asthma, mediate the generation of T-cell immune responses to dust mites and external microbial components, and together, they are involved in the development of asthma (13).

#### 2.2.2 pDCs

Plasmacytoid dendritic cells (pDCs) are produced continuously in the bone marrow. They are secreted and released in the peripheral area upon maturation, remain nonproliferative, and have a relatively short lifespan (23). Upon activation, MHCII expression is upregulated on pDCs (11). Additionally, Their endosomes are equipped with high levels of the nucleic acid-sensitive Toll-like receptors, TLR7 and TLR9 (24), which recognize single-stranded RNA and unmethylated CpG motif-containing DNA, respectively. pDCs secrete large amounts of IFN-I, IFN-III, cytokines (e.g., TNF-α), and chemokines in response to exposure to nucleic acids (25). In mice, pDCs are defined as bone marrow, lineage-negative, low-density cells in the blood and lymphoid organs, expressing markers such as CD11c^int^ CD45RA^hi^ CD45R^hi^ CD317^+^ MHCII^lo^ CD172^+^ CD11b^-^. They also express significantly high levels of Ly6C, Ly49Q, and Siglec-H (14, 15). In human pDCs express lineageneg CD11c^lo^ CD123^+^ HLA^-^DR^+^ BDCA2 (CD303^+^) BDCA4 (CD304^+^) CD45RA^+^ (15, 16). To summarize, mouse pDC differs from cDC in that pDC expresses Siglec-H, B220 (CD45R), Ly6C, and PDCA1 (CD317) and moderate levels of CD11c, whereas, human pDCs express BDCA2 (CD303), BDCA4 (CD304), CD123 (IL-3R), CD45RA, and HLA-DR but not CD11c. Moreover, human pDC surface markers share some common expression indicators with mouse pDCs; however, unlike mouse pDCs, human pDCs do not express CD45R.

The functional aspects of pDCs are reflected mainly in the production of large amounts of type I IFN against viral nucleic acids, thus imparting antiviral immunity. It also protects against fungal-invasive diseases (26). Additionally, pDCs were used as a vaccine for killing tumors by promoting CD8^+^ T-cell-mediated killing (27). However, whether pDCs are involved in the cross-presentation of exogenous antigens to CD8^+^ T cells is not clear. Several studies have shown that mouse and human pDCs can cross-present antigens *in vitro* (28, 29). However, some findings also suggest that pDCs are not involved in cross-presentation *in vivo* (30, 31). Besides being involved in the protective immune response, pDCs are also involved in protective immune tolerance. For example, upon inhalation of antigens or particles, pDCs generate immune tolerance to protect epithelial cells from damage caused by aberrant T-cell responses to otherwise harmless antigens and particles (32). Several experimental studies have shown that pDCs are strongly associated with asthmatic disease, alleviating the inflammatory response of the lungs of asthmatic mice. Moreover, depletion of pDCs exacerbates inflammation (33, 34).

#### 2.2.3 MoDC

Most studies on cDC2 localization have used CD11b (35, 36); however, CD11b is not only expressed by cDC2s but is also highly expressed in MoDCs (13). MoDCs are a subpopulation of inflammatory DCs that are produced in response to inflammatory stimuli, with enhanced recruitment under inflammatory conditions. They are present in various lymph nodes and tissues, such as the skin, lungs, and colon (37, 38). MoDCs cannot be easily differentiated from cDCs. MoDCs share many common markers with cDC2s, including CD11b, CD11c, MCHII, and markers of MoDC heterogeneity. Under normal conditions, cDCs can be differentiated into CD103^+^DCand CD11b^+^DC (cDC1s and cDC2s, respectively); under inflammatory conditions, monocytes derived from mouse and human Ly6C^+^ or CD4^hi^ can differentiate into MoDCs that migrate to sites of inflammation in a CCR2/CCL2-CD12-dependent manner (39), whereas, CD11b^+^ DCs and CD103^+^ DCs do not depend on this process (40). MHCII^+^ CD11c^+^ CD11b^+^ CD64^int^ Ly6C^int^ CCR2^+^ CD209^+^ are defined as mouse MoDCs, and HLA-DR^+^ CD11c^+^ CD14^int^ CD206^+^ CD1c^+^ are defined as human MoDCs (6). Recently, the use of single-cell RNA sequencing to sequence DCs for clustering has emerged, in which MoDCs are assigned to three DC groups (41).

## 3 Pathogenesis of asthma

Asthma is a heterogeneous disease with significant differences in the age of onset, associated risk factors and severity, comorbidities, and response to therapy (42). Several studies have reported that type 2 (T2) inflammation is the essential immune response in asthma pathology (43). Asthma is categorized as high T2 and low T2 (non-T2) types based on clinical and pathophysiological characteristics (44).

### 3.1 T2-high asthma

Airway Epithelial Cell-Derived Cytokines Modulate Dendritic Cells, Participating in the Pathogenesis of T2-High Asthma. When airways are exposed to pathogens, allergens, and environmental pollutants, various pattern recognition receptors on airway epithelial cells, including cell-surface Toll-like receptors (TLRs) 1, 2, 4–6, and 10 (24), and C-type lectin receptors (CLRs), such as dectin-1, mannose receptor (MR), and protease-activated receptor (PAR-2), which are triggered by airway epithelial cells, leading to the release of chemokines and epithelial-derived cytokines, which in turn trigger a downstream inflammatory cascade of various immune cells and cells of subepithelial structures (45, 46).

**Figure 2:**
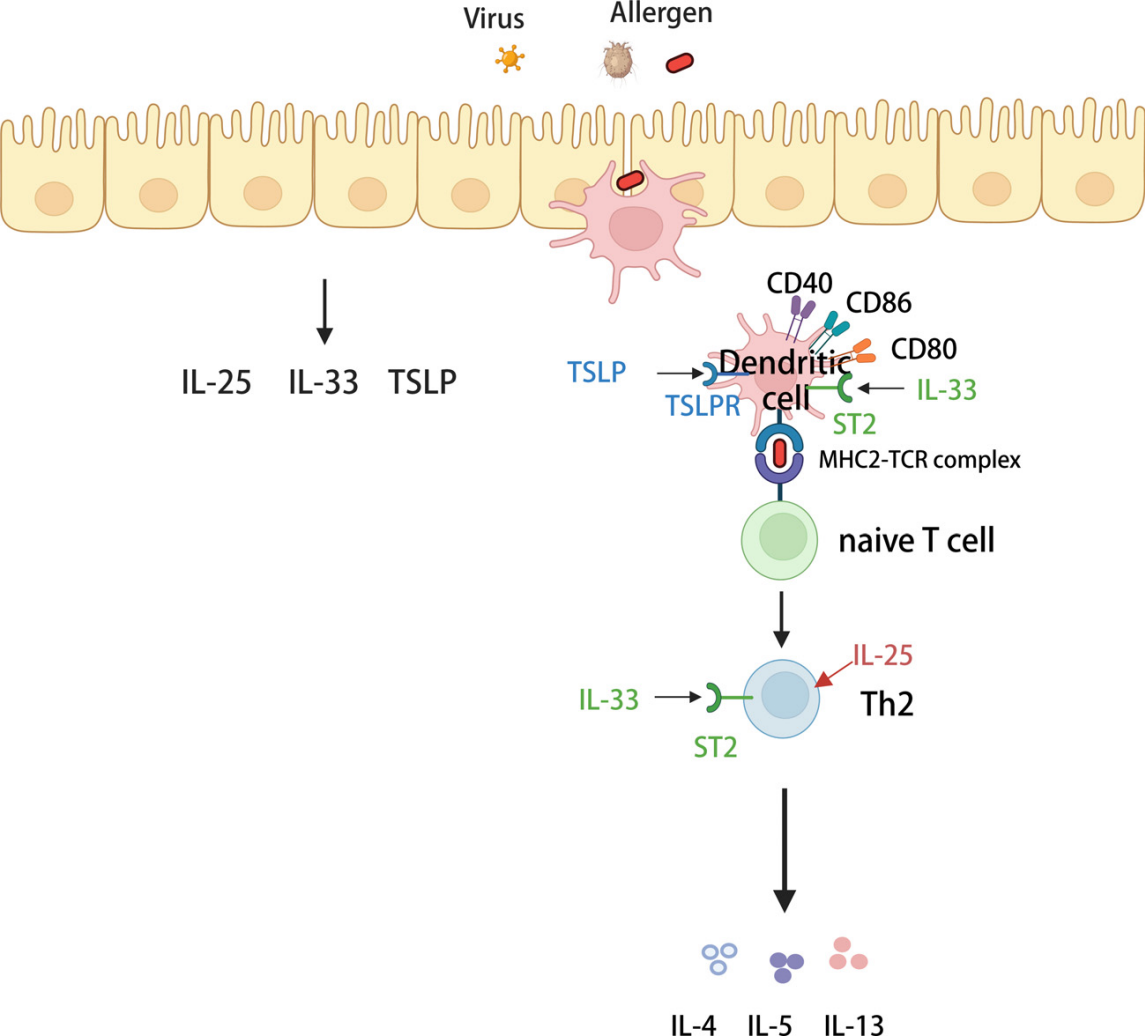
Mechanisms of T2-high asthma. Various allergens invade airway epithelial cells and stimulate airway epithelial cells to secrete IL-25, IL-33 and thymus stromal lymphopoietin (TSLP). Dendritic cells (DCs) recognize and then capture various allergens and carry them to regional *lymph nodes*, where they activate naive T cells. In the presence of specific cytokines, naive T cells differentiate into helper T cells 2 (Th2) and secrete type 2 cytokines (including IL-4, IL-5, and IL-13) involved in high-T2-type inflammation. Among them, three alarm*ins* (IL-25, IL33, and TSLP) can act on dendritic cells or Th2 cells to enhance the secretory production of type 2 cytokines.

The protease activity of antigens such as *Streptococcus* and HDM, as well as, fungal and viral infections, can cause the airway epithelium to secrete three epithelial-derived allergens, including IL-25, IL-33, and thymic stromal lymphopoietin (TSLP) (47), which promote or modulate Th2 immunity by directly or indirectly affecting DC function, ultimately mediating the inflammatory immune response in asthmatic airways. TSLP induces the maturation of DCs and upregulates their surface costimulatory molecules, including CD40, CD86, CD54, CD80, CD83, and CD-LMAP, to promote CD4^+^ T-cell proliferation and induce IL-4 gene transcription for the differentiation of Th2 cells (48, 49). IL-25 enhances the production of Th2 transcription factors in an IL-4-independent manner, which directly promotes the production of Th2-type cytokines by Th2 memory cells de-activated by TSLP-activated DCs (50). Additionally, IL-33 can indirectly regulate Tregs by inducing the production of IL-2 by DCs and ILC2s, thus promoting Treg proliferation to regulate inflammation (46). IL-33 not only regulates Th2-type immunity but also promotes Th2-type immunity. The IL-33 receptor, ST2, is expressed on CD4^+^ T cells, ILC2s, DCs, eosinophils, and other cells (51). IL-33 regulates Th2-type immunity by binding to the ST2 receptor on the surface of Th2 cells, which produces type 2 cytokines and promotes Th2-type immunity (52). Besnard et al. reported that the IL-33/ST2 pathway initiates DC *in vivo* against Th2 cells after exposure to antigens (53). Similarly, Rank et al. reported that dendritic cells respond directly to IL-33 via ST2 (54). Thus, IL-33 may also be involved in Th2-type immunity by indirectly promoting the polarization of initial T lymphocytes to Th2 cells through interactions with ST2 receptors on the surface of DCs. Along with the conventional three epithelial alarmins IL-25, IL-33, and TSLP, a novel epithelial alarmin member, HMGB1, has attracted attention (55). Ullah et al. demonstrated an increased frequency of MoDCs in Pag1-deficient mice and that HMGB1 may be involved in the progression of asthma by activating immature DCs, increasing their antigen-presenting capacity, and ultimately, promoting CD4^+^ T-cell differentiation to participate in the inflammatory response in T2-high-type asthma (56) (**Figure 2**).

### 3.2 T2-low asthma

**Figure 3:**
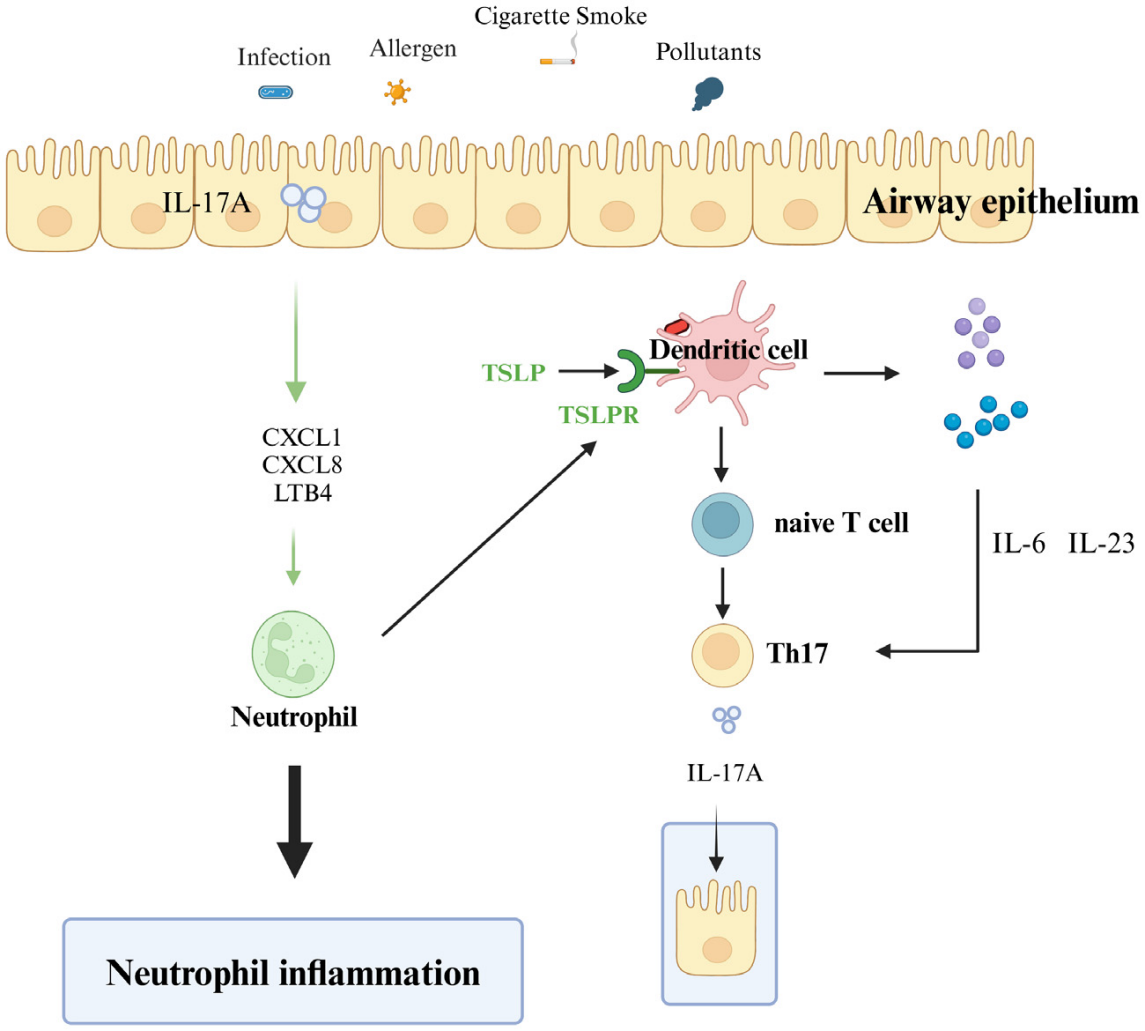
Mechanisms of T2-low asthma. Various external stimuli (including allergens, smoke, pollutants, and infections) stimulate and activate receptors on the surface of epithelial cells, and dendritic cells present antigens to promote the differentiation of naive T cells to helper T cells 17 (Th17). Th17 cells secrete IL-17A, and IL-17A acts on airway epithelial cells to produce neutrophil chemokines (e.g. CXCL1, CXCL8) and leukotrienes (LTB4), which recruit neutrophil inflammatory infiltration. The neutrophil cytoplasm of infiltrating airway epithelial cells induces dendritic cells to promote initial T-cell differentiation to Th17. Dendritic cells stimulated by antigens produce IL-6 and IL-23 and promote the differentiation and production of Th17 cells. Th17 produces IL-17A, which acts on airway epithelial cells and repeatedly exacerbates the inflammatory infiltration of airway neutrophils.

Although an increase in T2 inflammation occurs in two-thirds of individuals with asthma, 33% are classified as having T2-low asthma, a highly heterogeneous condition consisting of multiple T2-low asthma phenotypes (57). Next, we discuss the pathogenesis of neutrophilic asthma involving DCs (**Figure 3**).

Three epithelial cell-derived epithelial alarmins, IL-25, IL-33, and TSLP, are involved in the differentiation of Th17 cells. Upon stimulation with TSLP, DCs undergo activation and maturation, subsequently orchestrating the polarization of naïve T cells into the pro-inflammatory Th17 subset (58). Second, IL-33 is involved in the Th17-type immune response through the IL-33/ST2 axis, which regulates the secretion of IL-17A (59). Moreover, IL-17 is also associated with severe asthma and higher expression in patients with moderate-to-severe asthma (60). Airway epithelial cells can induce the differentiation of CD4^+^ T cells toward Th1 and Th17 immune responses after activation of TLR receptors (24, 61). Neutrophil inflammation is usually associated with IL-17 cytokines (IL-17, including IL-17A), and IL-17A is produced by Th17 cells. IL-17A induces the release of neutrophil chemokines (e.g., CXCL1 and CXCL8), leukotrienes (LTB4) involved in airway epithelial neutrophil recruitment chemotaxis, and neutrophil chemotaxis to sites of inflammation (62). Additionally, IL-23 and IL-6 produced by DCs after allergen stimulation further enhance the response of Th17 cells and maintain the continued production of IL-17 by Th17 cells (63). IL-17A, in turn, acts on airway epithelial cells, induces neutrophil chemokines, and creates a positive feedback loop that promotes further neutrophilic inflammation. Similarly, neutrophils secrete CXCL8, generating a positive feedback loop that promotes neutrophilic inflammation (64). Krishnamoorthy et al. also found that the number of neutrophils in the cytoplasm is closely related to the concentration of IL-17 in patients and that the cytoplasm of neutrophils in mice can trigger DCs to induce the differentiation of antigen-specific Th17 cells (65). Neutrophils are chemotactically recruited to airway epithelial cells, in turn inducing the differentiation of CD4^+^ T cells into Th17 cells through the action of DCs. The Th17 cells secrete IL-17A to the airway epithelium, thus forming a positive feedback loop that further exacerbates lung inflammation and infiltration of inflammatory cells in the airways.

## 4 DC and asthma

### 4.1 Association between asthma and DC subgroups

Myeloid DCs induce a Th2-type inflammatory response in the airways of patients with asthma in response to aeroallergen exposure, leading to eosinophil infiltration and disease progression (66). pDCs also produce high levels of antiviral type 1 interferon and Th1 effector molecules (67). However, pDCs may also play a role in generating tolerance to inhaled antigens to protect epithelial cells from damage caused by aberrant T-cell responses to other innocuous antigens and particles (32). Lung cDC1s in a mouse model of asthma were found to mainly promote Th1-type and Th17-type responses *in vitro*; cDC2 promotes Th2-type responses, and Th17-type immunoreactivity leads to the infiltration of inflammatory neutrophils associated with severe asthma (68, 69).

### 4.2 Role of different DC subgroups in the pathogenesis of asthma

#### 4.2.1 cDC1

Dendritic cells play a critical role in the immune response to inhaled allergens by ingesting the allergen, transporting it to the draining mediastinal lymph node (mLN), and presenting the antigen to initiate a CD4^+^ T-cell response. In the lungs, cDCs are divided into cDC1 and cDC2 (12). cDC1s are mostly regarded as bystanders, Compared to other DC subpopulations, cDC1s do not present obvious inflammatory immune characteristics (70). Not only is there no significant change in the number of cDC1 in the mouse model of asthma (71, 72), but there is also no increase in the number of cDC1s in the bronchial tissues of patients with asthma after the inhalation of allergens (73). This may be related to the role of cDC1s in immune function during inflammation.

##### 4.2.1.1 cDC1s induce Th1- and Th17-type immune responses in asthma and can suppress Th2-type responses by producing IL-12

A study revealed that in an ovalbumin (OVA)-induced asthma model, cDC1-induced CD4^+^ T cells secreted significantly more IL-4, IL-6, and IL-10, whereas cDC1-induced higher frequencies of IFN-γ and IL-17A production by CD4^+^ T cells. Additionally, The chemokines CXCR3 and CCR5 are preferentially expressed in Th1 cells, but their levels are also significantly high in cDC1-sensitized CD4^+^ T cells (68). These findings suggested that cDC1s primarily assist in the inflammatory response associated with asthma by triggering Th1 and Th17 responses, such as cellular immunity, while inhibiting the Th2 response by producing IL-12. In the helminth infection study conducted by Everts et al., DCs were harvested from both wild-type and Batf3-deficient mice (lacking cDC1 cells), It is well-established that cDC1 possesses the distinct capability to secrete IL-12. Subsequently, these DCs were challenged with ovalbumin (OVA) allergens and subsequently treated with either exogenous IL-12 or anti-IL-12 antibodies. The findings revealed that the DCs within the mLN of Batf3-deficient mice significantly enhanced Th2 polarization of T cells. Furthermore, it is noteworthy that cDC1 has been shown to secrete IL-12. While the study did not definitively confirm the role of cDC1 in activating Th1 and Th17 immune responses, it provided evidence that cDC1 can suppress Th2-type responses through the secretion of IL-12 during inflammation triggered by spirochete infection (74).

##### 4.2.1.2 cDC1s promote a Th2-type immune response by secreting associated cytokines

Eosinophilic infiltration is a hallmark of allergic asthma, and eosinophilic asthma, also known as T2-high-type asthma, is critical for Th2-type immune responses. Some studies suggest that cDC1s do not inhibit the Th2 response but instead promote the Th2 response during inflammation and play a vital role in T2-high-type asthma. Specifically, T cells co-cultured with cDC1s stimulated with OVA/HDM *in vitro* produced higher levels of Th2-associated cytokines IL-4, IL-5, and IL-13, resulting in the production of low levels of IFN-γ (68). *In vivo*, in HDM-exposed wild-type mice, CD103^+^ DCs in the lungs increased, and CD4^+^ T cells, CD8^+^ T cells, and Treg subpopulations all expressed CD103 (75). Significantly fewer mucus cells were produced in the airways of BXH 2 (cDC1-deficient) mice than in those of wild-type asthmatic mice, with lower levels of IL-4, IL-13, and IL-17 in the lungs (76). Second, Yi et al. demonstrated this idea by establishing an asthma model with OVA-induced wild-type and Batf 3-deficient (cDC1-deficient) mice. They reported that cDC1s can directly recruit eosinophils by secreting CCL17 and CCL22, which promote the development of T2-high-type asthma after stimulation by allergens in mice (77). These findings suggested that cDC1s are necessary for triggering the Th2 response to inhaled allergens, whereas they have a weak response or do not respond to Th1 and Th17 cells. This finding contradicts the previous view that cDC1s are involved mainly in Th1 and Th17 responses, predominantly in patients with asthma. Therefore, further in-depth studies are needed to address the specific role played by cDC1s in the pathogenesis of asthma.

#### 4.2.2 cDC2

Type 2 classical dendritic cells (cDC2s) play important roles in immune responses against allergens, viruses, fungi, and helminths and are recognized as the significant DC subpopulation involved in atopic asthma (78). During sensitization, DCs act as APCs to transport ingested allergens to the mLN, initiating an antigen-specific adaptive immune response involving T cells. In contrast, among DC subpopulations, cDC2s play an essential role in inducing a Th2-type immune response by antigen presentation in allergic pneumonia, and data show a significant increase in cDC2s in alveolar lavage fluid in a model of bronchial asthma (79).

##### 4.2.2.1 cDC2-dependent interferon regulatory factor 4 (IRF4) drives Th2- and Th17-type immune responses

The development of cDC2s largely depends on IRF4, and IRF4 expression in dendritic cells is positively correlated with enhanced antigen presentation (80). Moreover, IRF4 promotes the differentiation of naive T cell cells into effector Th cells, which play a key role in the immune response of cDC2s (81). Williams et al. found that after stimulation by HDM allergens, cDC2s rapidly induce IRF4 to promote IL-10 and IL-33 production to drive Th2-specific responses, and Th2 cells also promote stimulus-induced DC expression of IRF4, thus creating positive feedback (10). In allergic responses, colony-stimulating factor 1 (CSF-1), secreted by respiratory epithelial cells, regulates DC recruitment in an IRF4-dependent manner. It promotes the migration of cDC2s to local lymph nodes and secretes the Th2-type-associated cytokines IL-4 and IL-13 (79). Moreover, Schlitzer et al. reported that under inflammatory conditions, mouse CD11b^+^ DCs and human CD1c^+^ DCs are co-dependent on IRF4 to secrete Th17-cell-predominant cytokines interleukin 23 (IL-23) and IL-17 to direct the differentiation of Th17 cells (81).

##### 4.2.2.2 cDC2s induce Th2 and Th17 cell immunity via the uptake and direct presentation of allergens

In response to HDM stimulation, cDC2s capture allergens and act as APCs to present extracellular antigens directly to CD4^+^ T cells. Dectin-2 expressed on CD11b^+^ DCs promotes Th2 and Th17 cell differentiation and allergic airway inflammation (82). Some studies have evaluated the allergenic potential of a subpopulation of lung DCs isolated from HDM-treated donor mice and transferred to unsensitized mice. *In vivo*, upon activation in the airways, cDC2s in the transferred lungs were found to transport antigens to the lymph nodes and induce allergen-specific Th2 cell responses that subsequently promoted eosinophilic airway inflammation (83), whereas mice selectively deficient in cDC2s exhibited an attenuated Th2 response and did not develop any allergic inflammation (84). Furthermore, Balhara et al. have recently observed that the absence of Pentraxin 3 is associated with higher counts and augmented activity of CD11c^+^ CD11b^+^ DC in asthmatic mice relative to their wild-type counterparts (85). This correlation, in conjunction with their earlier findings, points to a role for cDC2 in intensifying the inflammatory features of asthma through the facilitation of a Th17-type immune response (86). Additionally, Qian et al.’s recent study has shed light on how IL-10, derived from B-cells, enhances allergic sensitization in asthma through the regulation of Bcl-3. This research parallels the finding that CD11b^+^ DC play a role in promoting Th2-type inflammation in asthmatic conditions (87).

The results of the abovementioned animal studies showed that cDC2s are essential factors mediating asthma; therefore, researchers speculated whether the same results can be obtained in the clinical setting. Studies have shown that changes in the number and phenotype of cDC2s are also closely related to patients’ clinical conditions. An analysis of samples of peripheral blood, alveolar lavage fluid, and bronchial tissues from asthma patients revealed that the number of cDC2s increased significantly in asthma patients after they inhaled allergens; this effect was especially prominent in patients with frequent asthma exacerbations (73). Additionally, the expression of OX-40L was considerably increased in cDC2s in asthma patients with no or few acute exacerbations. In contrast, asthma patients with frequent acute exacerbations expressed lower levels of PD-L1 (a Th2-type immunosuppressant), suggesting that the frequency and phenotype of cDC2s can be differentiated between patients who exhibit a low or high number of exacerbations per year. These findings provide new ideas for the clinical differentiation of the severity of asthma (88).

#### 4.2.3 pDCs

Plasmacytoid DCs (pDCs) are derived from bone marrow-derived hematopoietic progenitor cells that circulate in the bloodstream and return to secondary lymphoid organs and sites of inflammation. Under certain circumstances, pDCs are the primary cells involved in antiviral immunity and exhibit potent proinflammatory or tolerogenic functions. Several subpopulations of pDCs have been identified, including CCR9(+), CD9(+), and CD2(+) pDCs. Three additional subpopulations of pDCs were recently identified, including the CD8α^-^β^-^, CD8α^+^β^-^, and CD8α^+^β^+^ subpopulations. These three phenotypes of pDCs play different roles in asthma, with CD8α^+^β^-^ and CD8α^+^β^+^ pDCs contributing to tolerance by inducing regulatory T cells in experimentally induced allergic asthma, whereas CD8α^-^β^-^ pDCs can induce and potentiate allergic lung inflammation (89, 90).

##### 4.2.3.1 pDCs: immunogenic drivers in asthma pathogenesis

Compared to cDCs, pDCs have a lower antigen-processing capacity and a unique function (91). In the immune response, pDCs secrete abundant interferon (IFN) and other immunoregulatory cytokines, which can sense pathogen invasion and regulate the immune response by secreting IFN-I (mainly IFN-α_1-13_ and IFN-β) (92) and activate other immune cells to respond to allergic reactions. They also stimulate the activation of CD4^+^ and CD8^+^ T cells and are involved in triggering naïve T cells and initiating adaptive immunity (93). In humans, pDCs are present in the sputum, and BALF of stable patients with asthma, and the number of pDCs is further increased by allergen triggering (94). pDCs are activated to take up inhaled allergens and migrate to the mLN to present allergens, while the expression of CD40 and OX-40L are upregulated to induce the activation of the Th2 cell response (95). In a murine asthma model, Wu and colleagues have demonstrated that neonatal mice exhibit heightened vulnerability to severe allergic airway inflammation after allergen exposure. This increased susceptibility is attributed to diminished pDCs counts, which in turn, skew the immune response towards a T2 bias. The researchers discovered that pDCs play a protective role by dampening allergic inflammation, achieving this modulation through a pDC-derived IFN-α-mediated mechanism that curbs the type 2 immune response (96).

##### 4.2.3.2 Plasmacytoid dendritic cells: key regulators of immune tolerance in asthma

Some studies have shown that pDCs exercise immune tolerance in asthma, and experiments have demonstrated that pDCs can protect against allograft rejection and autoimmunity by inducing regulatory T cells that mediate peripheral and central tolerance (97). In the OVA-induced asthma model, we observed that pDCs induce the differentiation of Treg cells, which in turn suppress antigen-specific T cell proliferation. Conversely, transient depletion of pDCs results in the expansion of dysfunctional Tregs, accompanied by immunoglobulin E sensitization, airway eosinophilia, goblet cell hyperplasia, and Th2-cell cytokine production. These findings suggest that pDC depletion could potentially precipitate allergen-induced asthma. Furthermore, sustained overtransfer of pDCs prior to sensitization in a murine asthma model has been shown to significantly alleviate airway inflammation and Th2-mediated inflammatory responses. The exploration of these mechanisms offers novel insights into the prevention of allergic asthma in humans (32, 98).

##### 4.2.3.3 pDCs: pivotal mediators in asthma deterioration

Emerging research suggests a role for pDCs in intensifying asthma symptoms. Investigators have noted that while pDCs are consistently found in asthmatic patients, their numbers in sputum notably increase during acute asthmatic episodes. This elevation is linked to higher levels of inflammation and a more severe disease prognosis (99). Respiratory viral infections and type 2 (T2) inflammation are established as key determinants in the pathogenesis of asthma, with the potential to synergistically advance the disease’s trajectory (100). pDCs emerge as pivotal mediators, propelling the interplay between these two factors and thereby exacerbating asthma. As previously discussed, the secretion of IFN-α by pDCs is multifaceted; it engages in the immune response to allergic stimuli and is crucial in the antiviral response, manifesting its effects through a spectrum of downstream actions, notably the inhibition of viral replication (101). Furthermore, pDCs have the capability to express the high-affinity IgE receptor (FcϵRI). Notably, the expression of this receptor is significantly elevated in patients with allergic diseases when compared to healthy individuals (102). Gill et al. discovered that anti-IgE binding to FcϵRIa on pDCs was linked to reduced serum IgE and FcϵRIa expression, correlating inversely with pDC’s antiviral IFN-α response. Their use of the anti-IgE monoclonal antibody omalizumab to block this pathway successfully boosted IFN-α production, thereby suppressing type 2 inflammation and allergic reactions. Notably, the group with the most significant IFN-α response improvement showed the least asthma exacerbation, hinting that viral interactions with type 2 immunity could be pivotal in the clinical course of allergic airway diseases (103).

As our comprehension of pDCs deepens, particularly their growing role in asthma pathogenesis, targeted therapies against pDCs have emerged, showing promise in bolstering host defense against viral infections and in regulating airway inflammation. Research has indicated that IFN-λ potently suppresses Th2 responses by countering IL-4’s effects on CD4^+^ T cells and curbing the release of other cytokines linked to T2 inflammation (104). Further research is imperative to uncover alternative pathways through which pDCs might influence CD4^+^ T cell reactions in asthma. This endeavor could pave the way for harnessing pDC functions to develop more impactful clinical strategies for diagnosing, predicting, and treating acute asthma exacerbations.

#### 4.2.4 MoDC

Monocyte-derived DCs (MoDCs) are widely present in tissues and inflammatory environments. It captures, processes, and presents antigens while expressing high levels of MHC and costimulatory molecules, which are essential for the activation of T-cells and adaptive immune responses against specific pathogens (105). MoDCs primarily perform proinflammatory functions and contribute significantly to the immunopathology of asthma by producing chemokines.

##### 4.2.4.1 Recruitment of MoDCs drives airway inflammation in asthma patients

Research has revealed that the concentration of MoDCs in the sputum and alveolar lavage fluid is markedly elevated in asthmatic individuals when compared to healthy subjects (106, 107). Conversely, MoDC levels in the peripheral circulation are significantly reduced in asthmatics. Notably, a trend indicates that these levels further diminish with increasing asthma severity (108). This indicates that MoDCs are drawn from the bloodstream into the airways of asthmatics, playing a role in the disease’s pathogenic processes.

##### 4.2.4.2 MoDCs regulate inflammation through direct antigen presentation and intercellular modulation in asthma

In asthma, the primary function of MoDCs is to produce inflammatory chemokines during allergen stimulation and present allergens in the lungs, which act as inflammatory regulators in pulmonary allergic inflammation (109). MoDCs were recently shown to induce an anaphylactic Th2 response only when stimulated by high doses of HDM allergens (13). Thus, our hypothesis suggests that MoDCs may participate in a supportive regulatory role in the immune response to asthma. Furthermore, MoDCs play a role in the pathogenesis of asthma through intercellular regulation. Notably, In asthma patients, IL-33 is produced by activating TLR3 in MoDCs under the regulation of epithelial cells. MoDCs also upregulate the expression of TSLP and IL-17A, which activate CD4^+^ T cells to induce Th2-type and Th17-type immune responses, leading to allergic inflammation (110, 111). The CCL2 concentration in mouse BALF was found to increase in an OVA-induced mouse model of asthma, whereas CCL2 influx in the lung tissue of allergic mice was reduced after treatment with eggs of *Schistosoma mansoni*, which impaired the recruitment of MoDCs in the lungs, thus attenuating allergic responses (112). This advances our understanding and offers new avenues for exploring the role of MoDCs in the pathogenesis of asthma.

To summarize, MoDCs may serve as novel targets capable of fine-tuning the Th2 response in allergic asthma through their functions and improving asthma treatment. However, there are few studies on the mechanism of action of this cell subpopulation, and given the plasticity of MoDCs and their environment-dependent nature, ongoing research may address only a part of the function of this cell subpopulation. Thus, more studies need to elucidate the specific mechanism by which MoDCs influence asthma.

## 5 Asthma treatment studies with participation of DCs

Dendritic cells play a key role in the pathogenesis of asthma, especially in terms of the activation and maturation of DCs, which can influence the direction of differentiation of CD4^+^ T helper cells (6). The pathogenesis of asthma is closely related to various types of helper T lymphocytes (113). Many of the findings related to DC-related asthma therapy remain at the theoretical level. We summarized and discussed recent findings on the use of DCs in asthma treatment.

### 5.1 Novel biologics for targeted therapy

The five leading biologics approved for clinical use in the treatment or mitigation of asthma are anti-IgE (omalizumab), anti-IL-5/anti-IL-5R (mepolizumab and rilizumab), anti-IL-4R (dupilumab), and anti-TSLP (tezepelumab) monoclonal antibodies (43). Notably, omalizumab therapy led to a decrease in FcϵRIα expression on pDCs and diminished IgE cross-linking, thereby mitigating the progression of asthma through the enhancement of pDC-derived IFN-α responses (103). Tezepelumab effectively targets the TSLP locus by interrupting the critical interaction between TSLP and DCs, thereby influencing DC maturation. This intervention selectively modulates the differentiation of CD4^+^ T cells into Th2 or Th17 cells (49, 58). Moreover, preclinical trials are exploring the potential of itepekimab, which targets IL-33, and astegolimab, which targets the ST2 receptor of IL-33 (43). The drugs may influence the initial activation of DCs on Th2 cells *in vivo* following antigen exposure by effectively blocking the IL-33/ST2 pathway (55). OX40L, a co-stimulatory molecule found on antigen-presenting cells such as DCs, plays a pivotal role in immune responses (114). Amlitelimab, which targets OX40L, is a therapeutic candidate that may modulate DC activation and promote the differentiation of CD4^+^ T cells. This approach is currently under investigation in preclinical trials (43). In conclusion, the precise targeting of these biologics exerts therapeutic benefits in asthma by influencing the activity of dendritic cells.

### 5.2 Additional Experimental Studies

#### 5.2.1 DC-induced differentiation of CD4^+^ T cells to generate Treg cells mediates airway inflammatory regulation

Regulatory T lymphocytes in the immune system are directly involved in the inflammatory regulation of asthma. They prevent or modulate asthmatic airway inflammation through the effects of the upstream factor DC on the production of Treg cells.

Oral probiotics can induce the production of regulatory DCs, which further induce CD4^+^ CD25^+^ Foxp3^+^ Treg cells to regulate airway inflammation (115). Additionally, two drugs, including synthetic glutaraldehyde-crosslinked mannoprotein antigen-encapsulated OVA (MDO) and *Staphylococcus succinus* strain 14BME20, which are isolated from traditional high-salt fermented soy food dashi, can inhibit inflammation by inducing the development of immune tolerance in DCs, which, in turn, can increase Treg cell production (116, 117).

These studies are based on conclusions drawn in the OVA-induced asthma model. OVA is not a common allergen in human allergic asthma. Besides OVA, HDMs also act as common allergens in patients with clinical asthma, and all of these induced models have been widely used as powerful tools to study the pathogenesis of asthma. Due to the intrinsic properties of different allergens, some studies have yielded conflicting conclusions, and OVA-induced asthma models have been questioned in recent years (118). Further studies are needed to demonstrate the reliability of the findings of the abovementioned therapeutic and mechanistic studies in OVA-induced asthma models. Raspe et al. found that the immunomodulator *Helicobacter pylori*-derived molecule vacuolating cytotoxin A (VacA) exerts HDM-induced suppression of allergic airway inflammation by modulating DC-induced production of Treg cells (119, 120).

#### 5.2.2 Regulation of DC maturation mediates airway inflammation regulation

Various substances and therapeutic approaches modulate airway inflammation by inhibiting the maturation of DCs in asthmatic mice. Lin et al. treated mice in an OVA-induced asthma model with conventional therapy for rheumatic diseases by administering atractylodin (ATL) and reported that ALT can regulate the maturation of DCs and modulate the development of inflammation by decreasing the activation of DCs in response to OVA-specific T cells (121). Additionally, treating asthmatic inflammatory mice with the Chinese herbal component amide alkaloid (EB-A) resulted in the alleviation of inflammation. Administering EB-A to treat mice with asthmatic inflammation resulted in the alleviation of inflammation and inhibition of DC maturation (122). Kim et al. reported that secondary metabolites of aster healing-tissue-derived extracellular vesicles (AYC-EVs) ameliorated symptoms of asthma in mice via a mechanism of action similar to that described above for ALT, which also affects the maturation of DCs (123).

Along with the above conventional drug components, several microbial components involved in asthma pathogenesis, such as the gram-negative bacterial membrane LPS, have been identified (124). Min et al. reported that inflammation in allergic asthmatic mice treated with lipopolysaccharide-activated bone marrow-derived dendritic cells (DClps) was effectively alleviated, and DC surface molecules were reduced (7). Similarly, the oral administration of three different mixed strains to asthmatic mice resulted in a decrease in costimulatory molecules on the surface of DCs, impairing the ability of DCs to present antigens to T cells and promote the differentiation of CD4^+^ T cells (125). Thus, microbial constituents also surfaced again, and they can exert a pharmacotherapeutic effect by either inhibiting or decreasing the maturation of DCs.

#### 5.2.3 DC migration and asthma regulation

Studies on therapeutic drugs and methods for asthma have shown that asthma is modulated in part by influencing the migration of DCs that ingest antigens in the mediastinal lymph nodes.

Interfering with or inhibiting the migration of DCs can help control the symptoms of asthma. Jaiswal et al. reported that the small-molecule drug dimethyl fumarate (DMF) can alleviate the Th2-type immune response by interfering with the migration of DCs to mediastinal lymph nodes (126). Their study further demonstrated that DMF also modulates inflammation in immune-mediated allergic airway inflammation in mice, suggesting that DMF may have a pharmacotherapeutic role in airway inflammatory diseases, providing a scientific rationale for anti-asthma therapy. Maes et al. reported that airway inflammation in mice was controlled and alleviated after deletion of the gene encoding the STE20 kinase TAOK3 or following the loss of its kinase activity. This effect was demonstrated explicitly by the blockage of allergen HDM-induced migration of lung DCs to draining lymph nodes and the alleviation of airway inflammation (127). To summarize, exogenous pharmacological treatments and endogenous genetic interventions suggest that alleviating and controlling asthma symptoms may be possible by influencing the migration of DCs.

## 6 Conclusion and outlook

Different DC subpopulations play different roles in asthma. The cDC2 subpopulation is associated with promoting airway inflammation in HDM-induced or OVA-induced asthmatic mice in most cases, in contrast to the cDC1 subpopulation, which performs an inflammatory modulatory function. MoDCs, which are obtained from monocyte-derived proliferation and differentiation *in vivo* upon allergen stimulation, promote the progression of inflammation in the body via cDC2s. In DC-focused therapeutic research, including biotherapies and recent treatments with traditional drugs, chemicals, and bacteria, all influence DCs, thereby regulating asthma symptoms through effects on DC migration, maturation, and differentiation.

To elucidate the mechanism of action of different DC subgroups in asthma, several experimental studies have determined the roles of each type of DC in asthma. The current study’s findings predominantly highlight cDC2’s role in inducing Th2-type immune responses. However, additional data are necessary to determine the involvement of the cDC1 subpopulation in asthmatic Th2 responses. Second, while pDCs were once seen as mainly immune-tolerant, new data show they also have pro-inflammatory effects in asthma. Further research is needed to determine if pDCs have more roles in asthma beyond their known IFN-λ secretion, which counteracts IL-4’s impact on CD4^+^ T cells. This could help leverage pDC functions for better clinical management of asthma exacerbations. Regarding research on DC-related treatment, the therapeutic value can be further evaluated based on existing animal studies. Most notably, molecular compounds that can affect DC function can be screened to explore their therapeutic potential in asthma further. Ultimately, more effective medications need to be developed for the precise treatment of asthma patients with complex conditions and poor drug responsiveness.
